# Pilot Study of Diagnostic Potential of the *Mycobacterium tuberculosis* Recombinant HBHA Protein in a Vaccinated Population in Finland

**DOI:** 10.1371/journal.pone.0003272

**Published:** 2008-09-25

**Authors:** Laura Savolainen, Liana Pusa, Hwa-Jung Kim, Heidi Sillanpää, Ilkka Seppälä, Tamara Tuuminen

**Affiliations:** 1 Department of Bacteriology and Immunology, Haartman Institute, University of Helsinki, Helsinki, Finland; 2 Länsi-Uusimaa Hospital, Tammisaari, Finland; 3 Department of Microbiology, College of Medicine, Chungnam National University, Daejeon, South Korea; 4 Division of Clinical Microbiology, Helsinki University Hospital, HUSLAB, Helsinki, Finland; McGill University, Canada

## Abstract

**Background:**

In recent years T cell based interferon gamma release assays (IGRA) have been developed for immunodiagnosis of *M. tuberculosis* infection. At present these assays do not discriminate between disease and latency. Therefore, more promising antigens and diagnostic tools are continuously being searched for tuberculosis immunodiagnostics. The heparin binding hemagglutinin (HBHA) is a surface protein of *M. tuberculosis* which promotes bacterial aggregation and adhesion to non-phagocytic cells. It has been previously assumed that native, methylated form of this protein would be a promising antigen to discriminate latent from active infection.

**Methodology and Principal Findings:**

We performed a pilot investigation to study humoral and T-cell mediated immunological responses to recombinant HBHA produced in *M. smegmatis* or to synthetic peptides in patients with recent or past tuberculosis, with atypical mycobacteriosis, or in healthy vaccinated individuals. The T cell reactivities to HBHA were compared to the respective reactivities towards Purified Protein Derivative (PPD) and two surface secreted proteins, ie. Early Secretory Antigen Target-6 (ESAT-6) and Culture Filtrate Protein-10 (CFP-10). Our pilot results indicate that methylated recombinant HBHA induced a strong T cell mediated immune response and the production of IgG and IgM-class antibodies in all patient groups, most surprisingly in young Finnish vaccinees, as well. We observed a positive correlation between the reactivities to HBHA and non-specific PPD among all studied subjects. As expected, ESAT-6 and CFP-10 were the most powerful antigens to discriminate disease from immunity caused by vaccination.

**Conclusions:**

On the basis of results of this exploratory investigation we raise concerns that in countries like Finland, where BCG vaccination was routinely used, HBHA utility might not be sufficient for diagnostics because of inability to explicitly discriminate tuberculosis infection from immunoreactivity caused by previous BCG vaccination.

## Introduction

Newly introduced T cell based interferon gamma release assays (IGRA) are of a great diagnostic importance in distinguishing with high specificity the persons who are infected with *Mycobacterium tuberculosis* [1]. These assays, however, do not discriminate between disease and latent tuberculosis infection (LTBI) [2]. This limitation lowers their clinical utility e.g. in aging persons originating from a country with a previously high tuberculosis burden who present with pulmonary infiltrates or other manifestations suggestive of tuberculosis reactivation. Therefore, more promising antigens or diagnostic algorithms are continuously being searched to improve the current armament for tuberculosis (TB) immunodiagnostics. *Mycobacterium tuberculosis* heparin binding hemagglutinin (HBHA) is a virulence factor that promotes bacterial aggregation, adhesion to the heparan sulphate proteoglycans of nonphagocytic cells, and dissemination of tubercle bacilli from the lungs to other tissues in patients suffering from tuberculosis [3], [4]. Locht et al. [3] found that latently infected humans mount a strong Th1-type immune response to HBHA, whereas patients with active disease do not. Moreover, patients with active tuberculosis may develop a strong humoral response to native methylated HBHA [5], [6]. The diagnostic utility of HBHA-based interferon gamma release assays (IGRAs) has been further evaluated in Belgium [7], a country with low tuberculosis (TB) incidence where bacille Calmette et Guérin (BCG) vaccinations are rarely used. In that work, HBHA-based IGRA was not influenced by prior BCG vaccination and was significantly more sensitive than Early Secretory Antigen Target-6 (ESAT-6)-based IGRA.

Because some earlier studies [5], [7] concluded that previous BCG vaccination does not influence the assay performance we wondered whether HBHA-based methods would retain their diagnostic potential also in Finland. Our country currently has a low incidence of TB (6,1/100 000 [8]) but our population bore a high TB burden as recently as four decades ago. Moreover, since early fifties till September 2006, 98% of the Finnish population have been vaccinated. The rationale for this exploratory investigation was to compare immunological responses to the recombinant methylated HBHA produced in *Mycobacterium smegmatis* [9], methylated synthetic peptides from HBHA, Purified Protein Derivative (PPD), and the proteins with the documented high specificity for *M. tuberculosis* infection ie. ESAT-6 and Culture Filtrate Protein Derivative (CFP-10). For this pilot study we enrolled a few tuberculosis (TB) patients with a variable degree of disease activity and healthy young and middle-age Finnish vaccinees who were practically free from any previous contact with *M. tuberculosis* and thus served as a valid control group.

## Methods

### Human subjects and disease definitions

For this study we enrolled the subjects as follows:

#### a) Active TB group

Those subjects in whom the symptoms of the disease started within one month, who were hospitalized, anti-tuberculosis treatment was commenced within 2–3 weeks and who had a recent positive bacteriological verification. In this group four presented with pulmonary tuberculosis, one with tuberculosis of gastrointestinal tract and one patient had tuberculosis of cervical vertebra. In these subjects the blood samples were obtained within the routine laboratory investigation.

#### b) Inactive TB group

This group consisted of outpatients who presented with no or mild symptoms and who were either partially treated for TB with PAS, streptomycin, and INH or thoracotomy in forties and fifties, in whom chest X-ray findings indicated lack of an active process. Patients with vertebral TB had destructive processes of their vertebra at early childhood. At adulthood their TB was confirmed by radiology and the thick needle aspiration biopsy that ruled out other diseases. At the time of investigation these patients did not receive anti-tuberculosis treatment. Culture or nucleic acid amplification analysis (NAA) were either negative or bacteriological analysis was not performed.

#### c) Disease control group

Four patients with pulmonary manifestations in whom non-tuberculous mycobacterial (NTM) infection was diagnosed by a positive culture isolation. At the time of investigation these subjects were outpatients and their symptoms were mild (mainly cough).

#### d) Healthy control group

Finnish-born University students (mean age 25 years) who on the interview did not admit any previous contact with a TB patient and thus served as an ideal negative control, and Finnish-born laboratory personnel (mean age 50 years), were enrolled. All subjects of this group were born after the routine BCG vaccination campaign was implemented in Finland. Repeated vaccinations were discontunued in Finland in 1990.

### Ethical considerations

From the subjects of the group *a)* a verbal informed consent was obtained. The verbal informed consent was sufficient because the patient–doctor relationship was earlier established (Dr. Pusa), the patients attended medical care for the routine doctor's check-up and venipuncture was a part of their current medical treatment. From the groups *b)*, *c)* and *d)* a written informed consent was obtained before venipuncture. This study was approved by the Ethical Committee of the University Hospital of Helsinki, Internal Disease Department (Drno. 232/E5/07).

### Sample processing

Whole blood was withdrawn and divided into portions. From one portion sera were prepared by conventional methods and frozen at −20°C until use. From another portion peripheral blood mononuclear cells (PBMC) were isolated by Ficoll Paque gradient (Amersham Biosciences Inc., Piscataway, USA) and frozen in the CTL (Cellular Technology Ltd., Cleveland, USA) media in a liquid nitrogen until use. The characteristics of the individuals enrolled in this study are presented in Table 1.

**Table 1:** Characteristics of the individuals enrolled in the study.

Patient groups	ELISPOT (n)	EIA (n)	Ethnicity	Age range (yrs)	Gender	Bacteriology	Diagnosis
Active TB	6	4	Finns, n = 3 Persons from endemic area, n = 3 (2 with extrap. TB)	31–82	Female 2/6	All bacterilogically confirmed: culture+ n = 5, of those, acid faststaining+ n = 3 NAA+ n = 1	Tuberculosis: Pulmonary n = 4 GI channel n = 1 Cervical vertebra n = 1
Inactive TB	5	9	Finns, n = 8 One person from an endemic area (extrap. TB)	41–83	Female 4/9	At the time of examination culture and NAA negative or bacteriological analysis was not performed	Partially treated for pulmonary TB n = 7 Partially treated/untreated TB of lumbar vertebra diagnosed radiologically n = 2
Atypical mycobacteria	4		All Finns	66–75	All Female	*M. avium* n = 2 *M. intracellulare* n = 1 *M. abscessus* n = 1	co-morbidity with asthma, COPD
Vaccinated subjects students laboratory personnel	7 8	4	All Finns	22–59 mean 25 mean 50	Female 6/7 8		

### Synthetic peptides

Twenty three 15-mer sequential peptides overlapping by nine amino acids and spanning the genomic sequence of the *M. tuberculosis* (H37Rv) HBHA-protein starting from 37th amino acid were synthesized (Proimmune, Oxford, UK; Alta Bioscience, Birmingham, UK). The average purity of the peptides was ∼73%. Three peptides from the protein C-terminus were chemically methylated at their lysine residues (see Legend of Fig. 3). The methylation was done on the PEPscreen synthesis platform. Fmoc-Lys(Me,Boc)-OH were pre-dissolved at a 0.5 M concentration, placed on the deck, coupled and deprotected in the same way as standard amino acids. Additionally, the peptides for serology were biotinylated at their N-terminus. The quality control for all peptides was accomplished by mass spectrometry analysis. The peptides were dissolved in sterile asetonitrile and stored at −20°C in aliquots of 1–4 mg/ml with ∼30% glycerol for serology and in PBS for the T-cell assays.

### Recombinant HBHA antigen

Recombinant methylated HBHA was expressed in *M. smegmatis* (rMtb-HBHA). The pMV3-38 plasmid that contained the full-length HBHA open reading frame was kindly provided by Dr.G. Delogu (University of Sassari, Sassari, Italy) [9]. The protein from the bacterial extract was primarily purified by phosphocellulose chromatography, i.e., cation exchange, because most mycobacterial proteins do not bind to this resin at pH 7.0, and then further purified by Ni-NTA chromatography [10]. This two-step purification of rMtb-HBHA was highly effective. Contaminating bands on PAGE gels stained with Coomassie blue were not observed when 5–10 µg of the purified protein was loaded. The purified protein was stored at −20°C in aliquots until use in serology and T cell analysis.

### T ELISPOT analysis

T-cell reactivity to ESAT-6, CFP-10 (the synthetic peptides were from T-SPOT ®-*TB* kit, Oxford Immunotec, Oxford, UK), PPD (Statens Seruminstitut, Copenhagen, Denmark) and rMtb-HBHA was assessed by Enzyme-Linked ImmunoSpot assay (Mabtech Inc. Cincinnati, USA). Peripheral blood mononuclear cells (PBMC) were purified from fresh blood samples by density gradient centrifugation and stored with CryoABC Kit Freezing media (Cellular Technology Ltd.) in liquid nitrogen until use. After cells were washed with RPMI (HaartBio, Helsinki, Finland) and resuspensed in the CTL Test Media (Cellular Technology Ltd.), the cell count was performed by blood count-analyzer (ADVIA-60 Closed Tube Automated Haematology System, Bayer, Germany). The cells were diluted at 2.5×10^6^ PBMC/ml in CTL Test Media (Cellular Technology Ltd.). 250 000 PBMCs/well were stimulated in ELISPOT-plates in the presence of synthetic peptide-pool (10 µg/ml each), PPD (10 µg/ml), rMtb-HBHA (25 µg/ml). For viability test 50 000 PBMC/well were stimulated with phytohaemagglutinin (PHA) (Oxford Immunotech). CTL Test Media was used as a negative control. The plates were incubated at +37°C with 5% CO_2_ for 48 h. Thereafter the plates were washed and the analysis was performed according to the manufacturer's instruction. The spots were counted the ELISPOT-reader (Biosys, Lyngby, Denmark) and the net values were calculated by subtracting the readings of the media control. Spot-sizes and the cytokine values were examined with the ELISPOT-reader, AID EliSpot Software Version 4.0 (AID GmbH, Strasburg, Germany). The viability control test for the HBHA synthetic peptides was performed by exposing PBMC to the CFP-10 peptide mixture with asetonitrile added at concentrations of 15% and 30% (vs. only 2.5% in the final peptide solution). The addition of asetonitrile did not violate the reactivity to the CFP-10 antigen (data not shown).

### IgG and IgM determinations

The presence of IgG- and IgM-class antibodies to synthetic peptides, rMtb-HBHA and PPD were determined by enzyme-linked immunosorbent assay (ELISA). Synthetic peptides from *Borrelia burgdorferi* VlsE-protein IR6 region and positive serum samples from Lyme disease patients were used as the method control [11]. 96-well microplates (Microlon high binding, Greiner, Frickenhausen, Germany) were coated either with streptavidin (Roche, Mannheim, Germany) in PBS, pH 7.5 (100 ng/well), or PPD in PBS, pH 7.5 (1 mg/well), or with rMtb-HBHA in 0.1 M bicarbonate buffer, pH 9.5 (250 ng/well) overnight at +4°C. The plates were blocked with 0.25% Human serum albumin (HSA) (Finnish Red Cross, Helsinki, Finland) in PBS for 1 h at +37°C and washed four times with PBS containing 0.05% Tween20. Biotinylated synthetic peptides (500 ng/well) and IR6 (200 ng/well) in PBS, pH 7.5 were incubated for 2 h at room temperature. Plates were washed as above and the human sera diluted 1∶100 in PBS containing 0.5% HSA, 10% FCS and 0.1% Tween20 and were incubated for 2 h at room temperature. After wash Alkaline phosphatase-conjugated anti-Human IgG or IgM antibody (Jackson ImmunoResearch, W. Baltimore, USA) was added at 1∶5 000 dilution in PBS containing 0.5% HSA and 0.1% Tween20. After 2 h of incubation at room temperature the plates were washed four times. 4-nitrophenylphosphate (Boehringer Mannheim, Germany), 1 mg/ml in 0.1 M diethanolamine-0.5 mM MgCl_2_ was added to each well and the reaction was stopped after 15 minutes with 0.1 M NaOH. The plates were read at 405 nm with iEMS Reader MF (Labsystems, Helsinki, Finland). IgM rheumatoid factor was controlled for all serum samples by immunonephelometric method at the HUSLAB, Unit of Immunology.

### Statistical analysis

The correlation was analyzed by the non-parametric Spearman test. The Receiver operating characteristic (ROC) curve analysis and the Area under the curve (AUC) with respective 95% confidence intervals (CI) for each antigen were calculated with the GraphPad Prism version 4.0 (GraphPad Software, Inc. San Diego, CA).

The amino acid sequences of the HBHA from *M. tuberculosis* and *M. bovis* were searched with the Entrez Protein search engine and then the sequences were aligned with the Needle program, EBLOSUM62-Matrix (EMBOSS, [12]).

## Results

### Cell-mediated immunity

**Figure 1:**
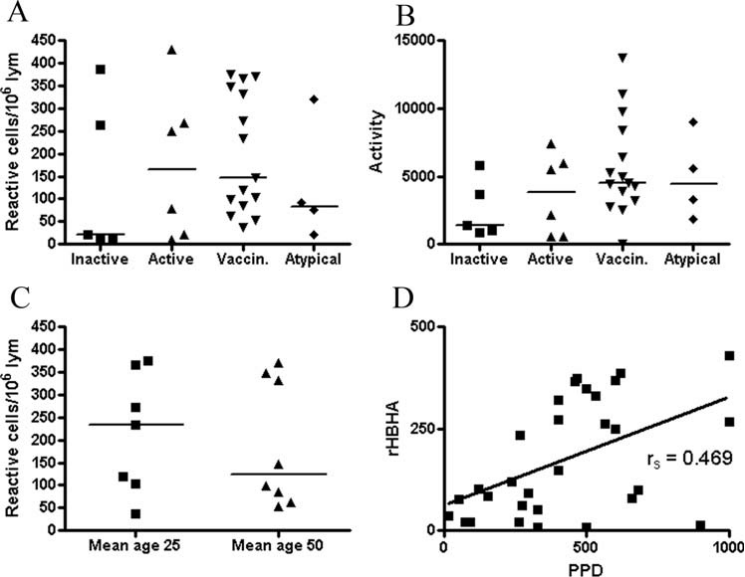
The ability of rMtb-HBHA and PPD to induce the production of IFNγ was tested in the ELISPOT technique. The groups of patients with inactive TB (n = 5); active TB (n = 6), healthy young (n = 7) and middle-age (n = 8) vaccinated subjects and patients with isolation of so-called atypical mycobacteria (n = 4) and were enrolled. Comparison of the cell-mediated immunological responses to rMtb-HBHA was performed as the determination of the number of reactive cells per10^6^ lymphocytes (A) and as the measurement of IFNγ production activities expressed as arbitrary units (B). Cell-mediated immunological responses in healthy vaccinated individuals was studied by division by age into two groups (C). The correlation of rMtb-HBHA with the PPD ELISPOT reactivities expressed as the frequencies of reactive cells per10^6^ is presented in (D). Data are shown as individual reactivities; the horizontal bars represent arithmetic median values.

Employing both techniques for immune reactivity study of rMtb-HBHA, we observed considerable interindividual variation in all studied groups (Fig. 1A–C and Fig. 3 A–B; D–F). These observations hold true irrespective of the measured parameters of immunoreactivity; neither the numbers of reactive cells, nor the amount of the IFNγ released, nor the optical densities in the IgG and IgM EIAs were discriminatory when comparing the groups. For example, the highest values of 430, 387, and 360 reactive cells/10^6^ lymphocytes were observed in the groups of active TB, inactive TB and healthy vaccinees, respectively. Strikingly, half of the vaccinated persons reacted strongly against rMtb-HBHA and the reactivities were equally high as those of the TB-patients (Fig. 1A). When the comparisons between the groups were done by semiquantitative calculation of the amount of the produced cytokine, ie. the size and the intensity of the spot, the highest median value of the cytokine production was found in the vaccinated group (Fig. 1B). Persons with positive environmental mycobacteria culture result reacted similarly as the persons from other groups with a high interindividual variation (Fig. 1A and B). Positive correlation was observed then the reactivities to rMtb-HBHA and PPD, an indicator of vaccination or infection, were compared for all the tested samples (Fig. 1D). One healthy 60-year-old individual who was not vaccinated in his childhood showed humoral and cell-mediated immune responses to rMtb-HBHA as measured by both EIAs and ELISPOT assay, indicating immunization with so-called atypical mycobacteria. Noteworthy, this person had a negative tuberculin skin test and his lymphocytes did not recognise ESAT-6 and CFP-10 peptide mixtures in ELISPOT (data not shown). Furthermore, the obtained reactivities were reproducible and the measurements did not exceed the expected between-run imprecisions of CV% ≤15 and ≤40% for EIAs and ELISPOT, respectively. When the frequencies of reactive cells were compared between persons of different ages, no differences were noticed either (Fig. 1C), indicating that there was no waning of immunological memory towards HBHA with age. In other words, ELISPOT analysis produced similar patterns of reactivities between the studied groups with 2 out of 5; 3 out of 6; and 7 out of 15 being strong reactors in the groups of inactive TB, active TB, and vaccinees, respectively (Fig. 1A).

**Figure 2:**
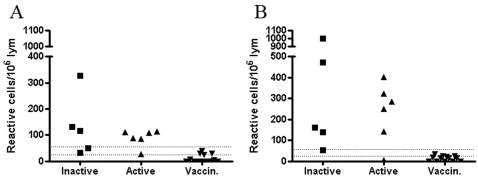
Production of IFNγ by lymphocytes stimulated with ESAT-6 (Panel A) and CFP-10 (Panel B) was tested in the ELISPOT technique. The groups of patients with inactive TB (n = 5), active TB (n = 6) and healthy vaccinated subjects (n = 13) were tested. Cell-mediated immunological responses were determined as the number of reactive cells per 10^6^ lymphocytes. The lower dotted line is the level of positivity suggested by the manufacturer, the upper dotted line is the level of positivity adopted at the HUSLAB diagnostic laboratory. The area between the two dotted lines represents the so-called grey-zone, an area of uncertainty for interpretation that was calculated based on assay imprecision (data not published).

As expected, when tested concurrently with a mixture of specific peptides derived from ESAT-6 and CFP-10 of *M. tuberculosis*, samples from patients with active and inactive TB did react in the ELISPOT assay, whereas samples from all healthy individuals, did not (Fig. 2 A and B).

### Humoral immunity to HBHA

**Figure 3:**
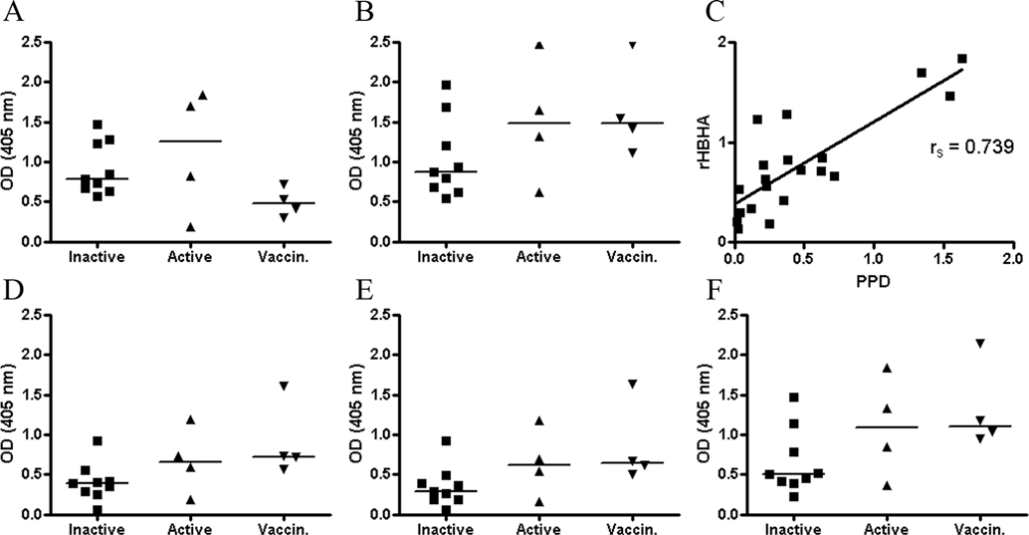
The serological reactivities to synthetic peptides, rMtb-HBHA and PPD were tested in IgG and IgM ELISAs. The groups of patients with inactive TB (n = 9), active TB (n = 4) and healthy vaccinated individuals (n = 4) were enrolled. Serological responses to rMtb-HBHA measured by the IgG EIA (A), IgM EIA (B), and IgG correlation in the PPD and rMtb-HBHA ELISAs (C). IgM responses to methylated 15-mer linear peptides from the C terminus of HBHA: IELPKKAAPA[KMe]KAAP (D), AAPAKKAAPA[KMe]KAAA (E), and AAPAKKAAPA[KMe][KMe]AAA (F). Individual responses are presented as optical densities (OD_405_). The horizontal bars represent arithmetic median values.

High interindividual variations were observed in serology, ie. 3 out of 9 (Inactive TB) and 2 out of 4 (Active TB) were strong reactors in the IgG and IgM EIA whereas almost all vaccinees showed moderate to high reactivities in IgG and IgM EIAs (Fig. 3A–B, D–F). Noteworthy, the median value of IgM antibodies to rMtb-HBHA was the highest in the group of healthy BCG-vaccinated individuals. Synthetic peptides were not recognised by any of the tested sera in the IgG EIA (data not shown). When tested for IgM rheumatoid factor it was detectable only in one person in the TB-patients group. Interestingly, IgM-class antibodies were detectable in almost all of the individuals in the study, with an exception of two non-vaccinated infants (data not shown). The IgM-class antibodies recognised not only rMtb-HBHA but also the three 15-mer linear methylated peptides from the HBHA C-terminus (Fig. 3D–F). We believe that IgM-class antibodies alone reacting to the C-terminal peptides of HBHA probably indicate a non-specific reaction arising from heterophilic antibodies, for example, or immunological cross-reactivity with conserved sequences of environmental bacteria (e.g., *rhodococcus*, http://www.ncbi.nlm.nih.gov/blast/Blast.cg).

### Comparison of the discriminatory power of rMtb-HBHA, PPD, ESAT-6 and CFP-10

**Figure 4:**
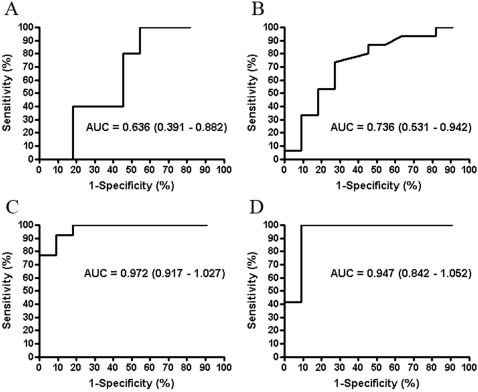
ROC curves were constructed to compare ELISPOT results when the cells were stimulated with rMtb-HBHA (A), PPD (B), and peptide mixtures of ESAT-6 and CFP-10 (C–D). The curves were established for infected (Active and Inactive TB) and the healthy control group. The calculated AUC and the respective confidence intervals (in brackets) are shown for each tested antigen.

ROC curves were constructed for all tested antigens and the AUCs with respective 95% CI were compared for each tested antigen (Fig. 4A–D). As expected, ESAT-6 and CFP-10 produced ROC curves acceptable for diagnostics with AUCs ranging from 0.947 to 0.972 and a narrow 95% CI (0.84–1.052) (Fig. 4C–D). On the contrary, the AUC for rMtb-HBHA (0.636; 95% CI 0.391–0.886) was comparable to the one for PPD (0.736; 95%CI 0.531–0.941) (Fig. 4A–B) indicating in practice no discriminatory power between healthy vaccinees and persons with a TB infection. As a consequence of high interindividual variation in rMtb-HBHA immunoassays, the confidence interval for the respective AUC was wide.

## Discussion

The potential of HBHA for diagnostics have been recently reported [3], [5], [6], [7], [10], [13]. In this pilot investigation we attempted to have an insight into the usefulness of this antigen for diagnostics in our fully vaccinated population. We aimed to compare immunoreactivity to rMtb-HBHA and HBHA methylated synthetic peptides with the immunoreactivity to the conventional PPD that have been used for decades with poor success because of inability to discriminate TB infection from immunity caused by vaccination. For comparison, we used two other secreted antigens, namely ESAT-6 and CFP-10. These antigens have been proven highly immunogenic and specific for *M. tuberculosis* infection and are absent from the majority of non-tuberculous mycobacteria and BCG substrain [for review see 1]. The major objective was to investigate whether immunological responses in persons with a proven contact with *M. tuberculosis* (Active and Inactive TB groups) would be quantitatively and qualitatively different from vaccinated individuals. In other words, we were interested to study the specificity of immune response to HBHA as a nominator of infection. The minor objective was to see whether immunological responses would differ in TB patients with a different degree of disease severity. We did not enrol a group of patients with a proven LTBI which may be considered as a limitation of our study. On the contrary, we enrolled young Finnish vaccinees that were unambiguously interpreted as free from LTBI and some patients with proven NTM infection. In our opinion, these two groups is a strength of our investigation. For analysis we used ELISPOT and EIA techniques.

The rMtb-HBHA antigen used in this study was prepared as described by Delogu et al [9]. Using a limited clinical material we were not able to affirm the discriminatory power of rMtb-HBHA in serology. Zanetti et al [6] who referred to the same method of the recombinant HBHA expressed in *M. smegmatis* [9], showed that only forty-four out of 111 sera with active TB produced optical densities over the presumed cut-off level of 0.5 OD_(405nm)_. In their study, surprisingly, the combined group of vaccinated people and patients with presumed latent tuberculosis infection did not produce IgG reactivities above the cut-off level. Using a larger cohort size than we did, Zanetti et al. [6] demonstrated a trend towards higher frequencies of responders in IgG serology in patients with active TB, however only 1/3 of the tested subjects were classified as serology positive. It seems however, that this frequency of positive results would not satisfy diagnostic needs. In our investigation we were able to detect IgM antibodies that recognized rMtb-HBHA and synthetic peptides of the C-terminus practically from all the tested individuals and also from one 60-year-old person who has never been vaccinated nor has a LTBI (data not shown). In the study of Shin et al. [10] who used immunoblot and EIA techniques to investigate IgM reactivity to rMtb-HBHA, the antibodies were detectable in early and chronic TB patients whereas healthy students, the controls, were non-responders. In their study, however, the vaccination status of the controls was not reported, therefore we are left with uncertainty how would this antigen be recognised in BCG vaccinees.

Masungi et al. [5] used HBHA that was purified from a BCG substrain. As others, they also reported that negative control subjects and the BCG vaccinees did not produce anti-HBHA antibodies at detectable level and the reactivity towards HBHA in lymphocyte stimulation assay was non-significant. On the contrary, when lymphocytes were stimulated with PPD, the controls and the BCG vaccinees reacted strongly [5]. In the most recent study of Hougardy et al. [7] HBHA was also purified from a BCG substrain and the cell-mediated immunity was studied as Masungi et al. by lymphocyte stimulation and a subsequent measurement of secreted interferon gamma (IFNγ). In their study the authors used control subjects who had no history of a TB contact but in whom half were BCG vaccinated. Quite opposite to our findings they observed the steepest ROC when lymphocytes were stimulated with HBHA while the stimulation with ESAT-6 produced the least discriminatory ROC.

Methylation of HBHA is crucial for effective T-cell immunity. Thus, native HBHA purified from BCG or *M. tuberculosis* H37Ra evoked a much stronger CD8+ and CD4+ mediated immune responses than the recombinant non-methylated HBHA produced in *E. coli* [13]. However, as indicated, recombinant *M. smegmatis* expression host was able to produce HBHA with a methylation pattern very similar to that of the native HBHA, as was evidenced by mass spectrometry analysis, amino acid sequencing, electrophoresis and recognition by the same monoclonal antibody [14]. There is however a possibility that folding of native HBHA produced by BCG or *M. tuberculosis* and of recombinant HBHA produced in *M. smegmatis* may be different which would cause minor differences in their immunoreactivities. The protein used in our study, rMtb-HBHA, was expressed in *M. smegmatis* and all attempts were made to avoid potential contamination that might have blurred the results. While it could be argued that minor *M. smegmatis* genome coded contaminant antigens might have caused the observed T cell responses in our study, the antibody responses (IgM) to the synthetic peptides support the view of weak specificity of the rMtb-HBHA responses in BCG vaccinated persons. In view of a longevity of immunological memory even without boosting [15], [16] it is not unexpected that our healthy vaccinees recognised rMtb-HBHA that has a 95.5% amino acid sequence homology to BCG HBHA [12].

Results of immunological studies with a battery of synthetic peptides comprising mono- and dimethylated peptides of the C- terminus of HBHA were very disappointing. In this study IgM-class reactivities were detectable but in a very unpredictable fashion while IgG-class antibodies did not recognize peptides even methylated ones in any of the patient groups. The peptides did not evoke any T cell mediated response. Noteworthy, our study protocol confirmed that the non-responsiveness to the peptides was not attributed to the toxicity of asetonitrile used in the peptides solution. Temmerman et al [13] studied T cell immunity with a peptide scan analysis first with non-methylated peptides and then probing a methylated peptide. Only one non-methylated peptide produced some IFNγ in a portion of LTBI patients. The reproducibility of this finding was not, however, reported. In their hands the methylated peptide induced also some IFNγ production but only in combination with the recombinant protein. The authors speculated that the methylated peptide might need a protein carrier. We too have no solid explanation why synthetic peptides behaved so differently in cell mediated and humoral immunity studies compared to the recombinant protein. In fact, we know yet little about how the HBHA is chopped in the lysosome and how the methylated antigens are presented by MHC II molecules.

Because our cohorts were too small to be able to pick-up minor differences in reactivity between the studied subjects we chose to use ELISPOT, the most sensitive and functional technique to study cell-mediated immunity. We assessed not only the frequencies of reactive lymphocytes (Fig. 1A) but the production of IFNγ as well (Fig. 1B). In this way we tried to do the analysis as close as feasible to that of Masungi et al. and Hougardy et al. However, in neither of the analyses could we prove HBHA superiority over the well-established antigens for diagnostics, namely ESAT-6 and CFP-10. As expected, these latter antigens possessed sufficient discriminatory power to separate infected individuals from vaccinated persons (Fig. 2A–B). The ROC analysis with a narrow 95% CI confirmed this conclusion (Fig. 4A–D). In fact, recent study by Chee et al. [17] showed that the two commercial methods for TB immunodiagnostics produced a poor agreement (κ = 0.257) when testing 270 patients with pulmonary TB. One was an ELISPOT- and the other was an EIA-based method. The authors speculated that the differences in results may be attributed to heterophilic antibody effects, non-specific IFNγ in the blood samples and lack of standardised lymphocyte counts in EIA-based technology compared to ELISPOT. The factors mentioned by Chee et al. [17] may also contribute to controversy between our pilot study and earlier studies [5], [7] where EIA-based methods have been applied.

This pilot study casts some concerns about the possibility to generalize about diagnostic potential of HBHA. We have observed that samples from healthy individuals in a country with almost complete vaccination coverage and even from individuals who have never been vaccinated may exhibit immune reactivities to HBHA, indicating that natural immunization to this protein or to cross-reactive peptides may occur. Therefore, on the basis of *i)* the presence of closely related antigen in a BCG substrain; *ii)* observed good correlation with PPD in serology and cell-mediated assays, *iii)* comparable ROC analysis for HBHA and PPD; *iv)* evidence of reactivities of vaccinees without any previous risk of contraction of *M. tuberculosis* infection, HBHA by no means is superior to ESAT-6 and CFP-10. In our environment HBHA practically does not add to tuberculosis immunodiagnostics. In conclusion, our results emphasize that the search for new more promising antigens for TB diagnostics should continue.
